# Non‐Cartesian GRAPPA and coil combination using interleaved calibration data – application to concentric‐ring MRSI of the human brain at 7T

**DOI:** 10.1002/mrm.27822

**Published:** 2019-06-10

**Authors:** Philipp Moser, Wolfgang Bogner, Lukas Hingerl, Eva Heckova, Gilbert Hangel, Stanislav Motyka, Siegfried Trattnig, Bernhard Strasser

**Affiliations:** ^1^ High Field MR Center, Department of Biomedical Imaging and Image‐Guided Therapy Medical University Vienna Vienna Austria; ^2^ Athinoula A. Martinos Center for Biomedical Imaging, Department of Radiology Massachusetts General Hospital, Harvard Medical School Boston Massachusetts; ^3^ Christian Doppler Laboratory for Clinical Molecular MR Imaging Vienna Austria

**Keywords:** 7 tesla, hybrid through‐time/through‐k‐space GRAPPA, MR spectroscopic imaging, multichannel coil combination, non‐Cartesian concentric ring, parallel imaging

## Abstract

**Purpose:**

Proton MR spectroscopic imaging (MRSI) benefits from *B*_0 _≥ 7T and multichannel receive coils, promising substantial resolution improvements. However, MRSI acquisition with high spatial resolution requires efficient acceleration and coil combination. To speed up the already‐fast sampling via concentric rings, we implemented additional, non‐Cartesian, hybrid through‐time/through‐k‐space (tt/tk)‐generalized autocalibrating partially parallel acquisition (GRAPPA). A new multipurpose interleaved calibration scan (interleaved MUSICAL) acquires reference data for both coil combination and PI. This renders the reconstruction process (especially PI) less sensitive to instabilities.

**Methods:**

Six healthy volunteers were scanned at 7T. Three calibration datasets for coil combination and PI were recorded: a) iMUSICAL, b) static MUSICAL as prescan, c) moved MUSICAL as prescan with misaligned head position. The coil combination performance, including motion sensitivity, of iMUSICAL was compared to MUSICAL for single‐slice free induction decay (FID)‐MRSI. Through‐time/through‐k‐space‐GRAPPA with constant/variable‐density undersampling was evaluated on the same data, comparing the three calibration datasets. Additionally, the proposed method was successfully applied to 3D whole‐brain FID‐MRSI.

**Results:**

Using iMUSICAL for coil combination yielded the highest signal‐to‐noise ratio (SNR) (+9%) and lowest Cramer‐Rao lower bounds (CRLBs) (‐6%) compared to both MUSICAL approaches, with similar metabolic map quality. Also, excellent mean g‐factors of 1.07 and low residual lipid aliasing were obtained when using iMUSICAL as calibration data for two‐fold, variable‐density undersampling, while significantly degraded metabolic maps were obtained using the misaligned MUSICAL calibration data.

**Conclusion:**

Through‐time/through‐k‐space‐GRAPPA can accelerate already time‐efficient non‐Cartesian spatial‐spectral 2D/3D‐MRSI encoding even further. Particularly promising results have been achieved using iMUSICAL as a robust, interleaved multipurpose calibration for MRSI reconstruction, without extra calibration prescan.

## 1 INTRODUCTION

Proton MRSI is a powerful noninvasive tool that enables in vivo imaging of local biochemical changes in healthy and diseased tissue.^1^, ^2^ The main obstacle for its clinical application is a low SNR, which ultimately limits the maximum achievable spatial resolution and minimum scan times.

The development of improved hardware (e.g., ≥7T MR scanners and multichannel receive coils)^3^, ^4^, ^5^, ^6^, ^7^ and more efficient data acquisition (e.g., short TE and direct FID detection)^8^, ^9^, ^10^ increase the SNR substantially. This SNR boost can be traded for impoved spatial resolution^8^, ^11^ or acquisition speed.^12^, ^13^, ^14^ Several recent MRSI studies provide evidence that high‐resolution 2D‐MRSI can be accelerated efficiently via PI at ≥7T,^12^, ^13^, ^14^ but also show that 3D‐MRSI – ideally whole‐brain – will be difficult to realize within clinically acceptable scan times without the use of faster spatial‐spectral encoding (SSE) techniques.^15^

The optimal combination of signals from all receive coil elements is a prerequisite, not only for high SNR efficiency, but also for good PI reconstruction. Coil combination is particularly challenging at higher magnetic field strength (B_0_) and for a larger number of receive channels.^16^ While non‐water‐suppressed MRSI techniques, such as metabolic cycling^17^ or SPICE methods,^18^ directly provide a water reference image, MRSI methods using water suppression require an additional – possibly interleaved – prescan.

The recently proposed MUSICAL method is based on a rapid imaging prescan, requires no reference coil, works with complete water suppression, is independent of MRSI spectral quality, and is particularly well tailored for FID‐MRSI, while providing the same coil combination efficiency as the gold standard of sensitivity map‐based approaches.^19^ Additional features of MUSICAL are an accurate 0‐order prephasing of all MRSI spectra and – similar to sensitivity map – MUSICAL intrinsically provides all the reference data necessary for k‐space‐based PI reconstruction.^13^ However, MUSICAL was developed for phase‐encoded MRSI, and, with the advent of non‐Cartesian SSE for the acceleration of ≥7T MRSI, the performance of the MUSICAL method needs to be revisited.

Long measurement times have mainly been tackled either by SSE techniques including spirals,^20^ echo‐planar spectroscopic imaging,^21^ rosettes,^22^ and concentric rings,^23^ or k‐space undersampling techniques, including GRAPPA,^24^ sensitivity encoding (SENSE),^25^ Controlled Aliasing In Parallel Imaging Results IN Higher Acceleration,^13^ and compressed sensing,^26^ Spatial‐spectral encoding offers high acceleration factors of up to two orders of magnitude^27^ but leads to increased demands on the gradient system, especially when simultaneously high spatial resolutions and spectral bandwidths are required at high *B*_0_. However, PI reconstruction efficiency benefits from higher *B*_0_, but is limited in its achievable acceleration. Acceleration factors of up to 10 have been repoted for PI,^13^ but at the cost of increasing spectral degradation mainly due to residual aliasing of lipid signals for higher accelerations.

Concentric‐ring SSE trajectories (one type of non‐Cartesian trajectories) have been described as most efficient at ≥7T to cover the increased spatial resolution and spectral bandwidth demands because of their self‐rewinding nature.^27^, ^28^ While the majority of MRSI reports employ only a single acceleration technique, even shorter scan times can be achieved by the simultaneous application of SSE and PI techniques that combine the benefits of both domains. Several MRSI reports have described the combination of proton echo planar spectroscopic imaging (PEPSI) with SENSE^29^, ^30^, ^31^ or GRAPPA.^32^, ^33^, ^34^ Spiral MRSI has been combined with iterative SENSE,^35^ an iterative k‐space based reconstruction,^36^ and random SENSE+TV.^37^ Concentric rings have been used for multiband‐SENSE in 3D‐MRSI,^38^ as well as in functional MRI combined with SENSE and GRAPPA.^39^

The purpose of our study was to demonstrate the first combination of non‐Cartesian concentric‐ring trajectories with a hybrid tt/tk‐GRAPPA reconstruction in an MRSI sequence. Through‐time/through‐k‐space‐GRAPPA has successfully been applied in radial cardiac MR imaging^40^, ^41^ and led to improved reconstruction quality compared to standard radial GRAPPA. Therefore, we developed an interleaved water reference scan that is used as calibration data for both coil combination and PI reconstruction, which capitalizes on all the benefits of MUSICAL but requires no prescan and is more general and, thus, could be tailored to arbitrary SSE trajectories. In contrast to MUSICAL, this new approach, termed interleaved‐MUSICAL, acquires the calibration data for coil combination and PI reconstruction in an interleaved fashion. This was motivated from MRI, where interleaved acquisitions have been described to reduce motion and scanner instability‐related erroneous PI reconstructions strongly.^42^, ^43^

## 2 METHODS

### 2.1 Volunteers and hardware

The study was performed on a 7T whole‐body MR scanner (Magnetom, Siemens Healthcare, Erlangen, Germany) with a 7T_SC72CD gradient system with 70 mT/m total gradient strength and 200 mT/m/s nominal slew rate. A 32‐channel receive coil array combined with a transmit volume coil (NovaMedical, Wilmington, MA) was used. Six healthy volunteers were included in this study. The study was approved by the Institutional Review Board. Written informed consent was obtained prior to MR examinations.

### 2.2 Calibration data acquisition

**Figure 1:**
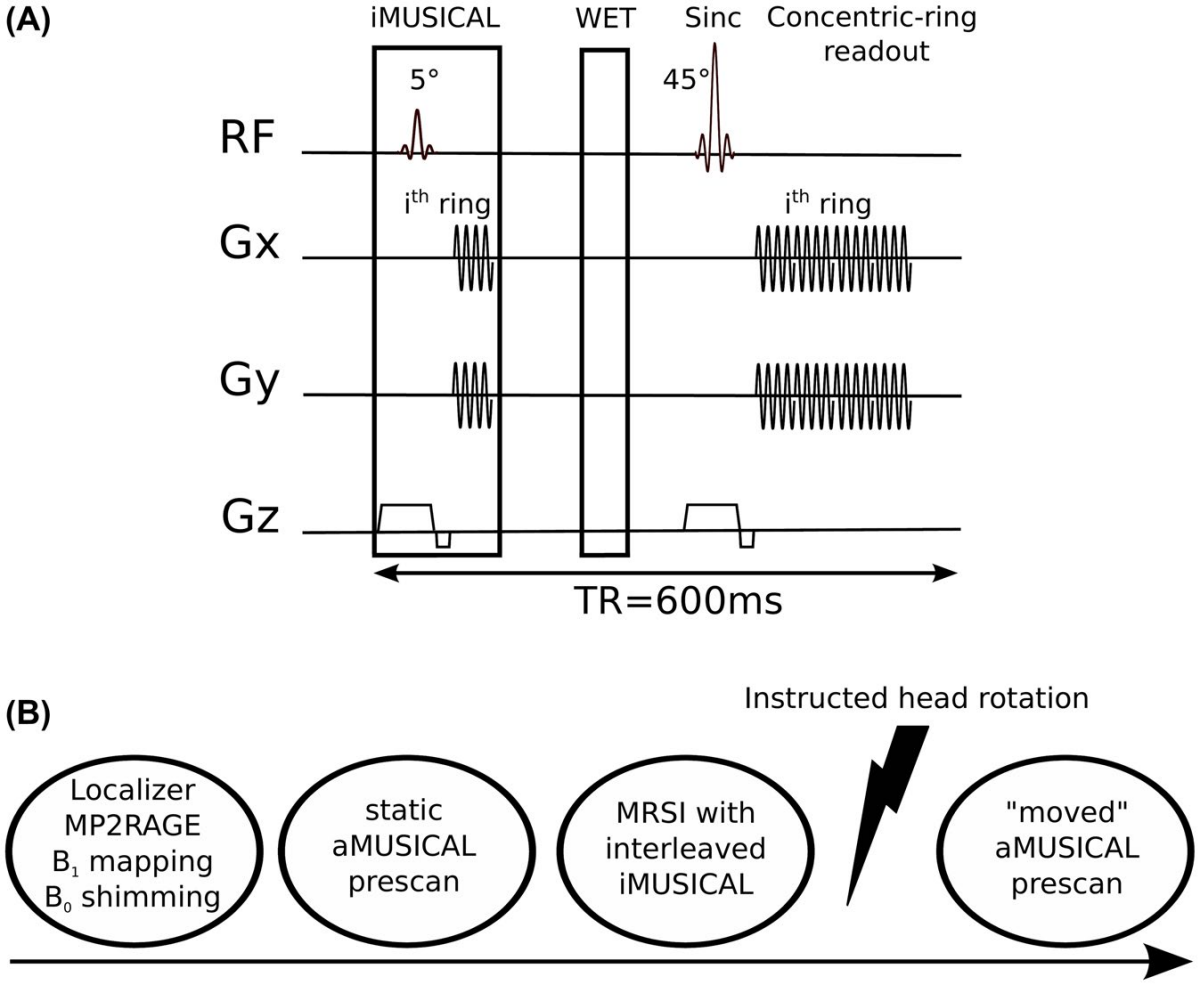
A, Schematic diagram of the single‐slice FID‐MRSI sequence: iMUSICAL prescan; water suppression enhanced through T_1_ effects (WET) and the single‐slice FID‐MRSI sequence with concentric‐ring readout (acquisition delay: 1.3 ms, TR: 600 ms). The extension to 3D‐MRSI was achieved by adding phase‐encoding in the partition direction; B, timeline of the measurement protocol FID‐MRSI, free induction decay‐MR spectroscopic imaging; TR, repetition time

Three different reference‐coil‐independent approaches for the acquisition of water‐unsuppressed calibration data (used for coil combination and PI reconstruction) were compared:

- Static adapted‐MUSICAL (aMUSICAL) method^19^: In the original paper, the coil sensitivities were approximated via a rapid Cartesian gradient echo imaging prescan before the MRSI scan. Its major scan parameters (i.e., echo time, field of view, matrix size) were matched to the subsequent phase‐encoded MRSI, but with a much shorter TR. For this study, the MUSICAL prescan readout had to be adapted to the non‐Cartesian concentric‐ring readout to match the MRSI readout, and thereby prevent possible incoherencies induced by differences in readout schemes (e.g., spatial response function). The head position was the same for the “static” aMUSICAL prescan as for the MRSI measurement. The acquisition parameters of the aMUSICAL prescan were identical to the MRSI scans (as listed in Data acquisition), except that only 27 spectral points were measured compared to 882 at a TR of 50 ms and a flip angle of 13° and no water suppression was used. The total scan times were 7 s and 1:53 min for a single‐slice acquisition (64 **×** 64 matrix) and a 3D scan (48 **×** 48 **×** 31 matrix), respectively.
- Moved aMUSICAL: Same as the preceding, but the volunteers were instructed to rotate their head (~15° left–right) after the MRSI scan and remain in this position during this prescan (Figure 1B). This allowed the assessment of motion artifacts in the coil combination and PI reconstruction when the mismatched calibration data were used.
- Interleaved‐MUSICAL: This new approach builds on the benefits of MUSICAL, but iMUSICAL directly integrates the required collection of calibration data in an interleaved fashion before the water suppression module within each TR of the “parent” MRSI sequence (Figure 1A). Thus, no additional scan time was required in our case as iMUSICAL only occupies dead TR fill time. This calibration module is identical to the MRSI acquisition module (i.e., in each TR, the same k‐space ring is measured in the iMUSICAL module as for the “parent” MRSI; acquisition parameters are listed in Data acquisition), except for a lower excitation flip angle of 5° to prevent noticeable saturation effects and only 27 spectral points. The residual transverse magnetization from the iMUSICAL module is spoiled before the MRSI excitation.

### 2.3 Data acquisition

All MR protocols included a 3D, T_1_‐weighted, magnetization‐prepared, two rapid acquisition gradient echoes sequence^44^ to position the MRSI slice. Field‐map‐based, first‐order and second‐order *B*_0_‐shimming using standard Siemens routines was performed over the volumes of interest of the individual MRSI scan. A *B*_1_^+^‐map^45^, ^46^ for pulse‐power calibration was acquired.

Five volunteers were scanned with a single‐slice FID‐MRSI (Figure 1A) sequence using the following settings: 600 µs sinc excitation pulse; *B*_1_, 13.2 µT; TR, 600 ms; acquisition delay, 1.3 ms; flip angle, 42°; slice thickness, 10 mm; field of view, 220 × 220 mm^2^; matrix size, 64 × 64; 32 equidistant rings (radius of first ring was half the Nyquist distance); points per ring, 540; complex spectral data points, 882; spectral bandwidth, 2778 Hz; receiver bandwidth, 500 kHz; temporal interleaves, 3; maximum used slew rate (x/y/z), 113.7/113.7/100 mT/m/ms; maximum used gradient strength (x/y/z), 20/20/20 mT/m; acquisition window, 317 ms; averages, 10; acquisition time, 9:52 min.

One volunteer was scanned with a volumetric 3D‐MRSI scan using the following settings: 600 µs sinc excitation pulse; *B*_1_, 13.2 µT; TR, 600 ms; acquisition delay, 1.3 ms; flip angle, 42°; field of view, 220 × 220 × 144 mm^3^; volumes of interest, 220 × 220 × 75 mm^3^; matrix size, 48 × 48 × 31; 3D‐stack of 24 equidistant rings each; points per ring, 540; complex spectral data points, 882; spectral bandwidth, 2778 Hz; apparent diffusion coefficient bandwidth, 500 kHz; temporal interleaves, 2; maximum used slew rate (x/y/z), 196.4/196.2/100 mT/m/ms; maximum used gradient strength (x/y/z), 21.8/21.8/20 mT/m; acquisition window, 317 ms; averages, 1; acquisition time, 14:59 min.

The “static” aMUSICAL prescan was acquired before the MRSI scan, while the “moved” one was acquired afterward (Figure 1B).

### 2.4 Data post‐processing

All measured data were processed with an in‐house‐developed pipeline based on Bash (Free Software Foundation, Boston, MA) and Matlab (MathWorks, Inc., Natick, MA) scripts.^47^ The postprocessing pipeline included a modified Pipe‐Menon pregridding density compensation,^48^ an off‐resonance correction and convolution gridding^49^, ^50^ using a Kaiser‐Bessel kernel (width of 3). Detailed steps are described in previous reports.^27^, ^47^

Spectral processing via LCModel 6.3^51^ was used to fit and quantify all MRSI data. In vivo spectra were fitted in a range of 1.8 to 4.2 ppm. The metabolite basis set consisted of 17 metabolite resonances, simulated via NMRScope,^52^ and a measured macromolecular background.^53^ glucose (Glc); aspartate (Asp); total choline (tCho) [glycerophosphorylcholine (GPC) + phosphorylcholine (PCh)]; total creatine (tCr) [phosphocreatine (PCr) + creatine (Cr)]; γ‐aminobutyric acid (GABA); Glx [glutamate (Glu) + glutamine (Gln)]; glutathione (GSH); glycine (Gly); lactate (Lac); myo‐inositol (Ins); tNAA [N‐acetyl‐aspartate (NAA) + N‐acetyl‐aspartyl glutamate (NAAG)]; scyllo‐inositol (Scyllo); and taurine (Tau).

### 2.5 Part 1 – coil combination

In this first step, no PI reconstruction was employed and the coil combination efficiency of iMUSICAL was compared to the two aMUSICAL cases for the fully sampled, single‐slice FID‐MRSI scans. These two aMUSICAL prescans obtained before and after the MRSI scan described previously (“static” and “moved”) allowed the assessment of motion artifacts that were introduced by a predefined head rotation.

To compare the different coil combination efficiencies, the spectral quality and fitting precision were evaluated. The spectral SNR was calculated for each voxel where the noise was calculated using an adapted pseudoreplica method, which – based on simple Monte Carlo techniques – rigorously calculates noise propagation through the reconstruction process even including reconstruction steps like GRAPPA.^54^ The signal for the SNR calculation was computed as the maximum signal in the tCr region of the LCModel‐processed spectra. The line width was calculated from the 3.02‐ppm tCr resonance as tNAA might be contaminated by lipid artifacts. The SNR, line width, and CRLB of metabolites (tCr, tCho, and tNAA) were assessed (mean, standard deviation, Cohen's effect size d) and compared among the three groups (iMUSICAL, “static,” and “moved” aMUSICAL). Instead of using all brain voxels, a mask of 20 randomly selected voxels was created and applied to each subject, such that each aforementioned group contained 100 spectra to avoid extremely large sample sizes in the statistical analysis. Kruskal‐Wallis ANOVA tests were used for intergroup comparisons, followed by Bonferroni‐corrected post hoc paired *t* tests. The total number of voxels where CRLB values of tCr, tCh, tNAA, and Glx were <20% was assessed in all cases.

### 2.6 Part 2 – hybrid through‐time/through‐k‐space GRAPPA

**Figure 2:**
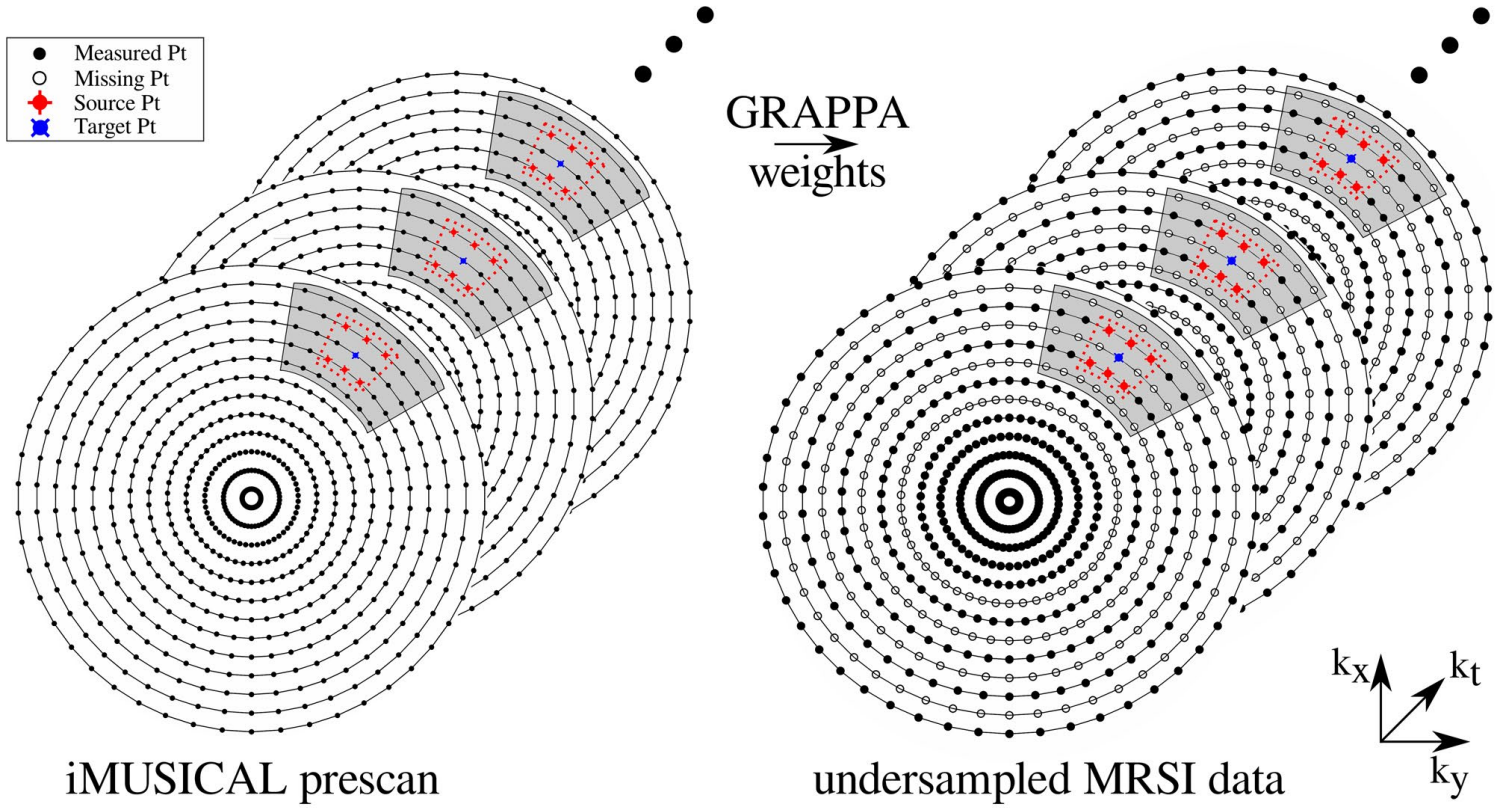
A schematic of the tt/tk‐GRAPPA method applied to concentric‐ring MRSI. The figure shows a fully sampled iMUSICAL calibration dataset on the left and a 2‐fold variable‐density undersampled MRSI k‐space on the right: the center of the k‐space is fully sampled (solid points), followed by constant 2‐fold undersampling (empty points). For calibration, a segment (shaded area) was used around each target point for the through‐k‐space GRAPPA part with a kernel size of 3 × 2 (red dotted lines), i.e., six source points. The kernel was slid through the segment of the calibration data to gather all the through‐k‐space kernel repetitions. Twenty‐one (21) calibration time points (through‐time kernel repetitions) were used. After calculating the tt/tk‐GRAPPA weights using the calibration data, the weights are used to reconstruct missing k‐space points in the undersampled MRSI data (right) MRSI, MR‐spectroscopic imaging; tt/tk‐GRAPPA, through‐time/through‐k‐space‐generalized autocalibrating partially parallel acquisition

Via GRAPPA missing k‐space points can be reconstructed from a linear combination of measured k‐space points. In Cartesian GRAPPA, the weights are assumed to be invariant to translations in k‐space, as long as the same pattern of measured and missing points is used. Therefore, the weights can be computed from a fully sampled calibration dataset. All possibilities ("kernel repetitions") to find the GRAPPA kernel in the calibration data are used to calculate the weights in a least‐square manner. This system of linear equations is overdetermined only if enough kernel repetitions are found. After calculating the weights from the calibration data, they are used to reconstruct the missing points in the undersampled data.^24^ Non‐Cartesian GRAPPA faces the problem that the translational invariance assumption in k‐space is violated. Therefore, non‐Cartesian through‐k‐space GRAPPA uses a segmented k‐space, where in each segment the translational invariance remains valid.^55^ In contrast, through‐time GRAPPA gathers the necessary kernel repetitions from different time points at the expense of longer measurement times for the calibration data.^56^ The hybrid tt/tk‐GRAPPA reconstruction^41^ combines both ideas and gathers kernel repetitions in k‐space and from multiple time points to determine the weights (Figure 2).

The undersampled MRSI data necessary for PI reconstructions were created in postprocessing by discarding the appropriate amount of rings from a fully sampled k‐space. In this way, a reference gold standard (i.e., the fully sampled k‐space) was available. Six undersampling patterns were simulated. For a constant two‐fold, four‐fold, eight‐fold undersampling, only every second, fourth, or eighth was kept, respectively, while the data of all remaining rings were discarded. In addition to this constant undersampling approach, a variable‐density undersampling approach was simulated: i.e., the innermost five rings were excluded from undersampling, while the outer k‐space was subject to a constant‐density undersampling (Figure 2). Previous MRI studies have shown that such a variable‐density undersampling approach significantly reduces artifact sensitivity.^57^, ^58^ The reconstruction quality using three different calibration datasets (iMUSICAL, “static,” and “moved” aMUSICAL) was compared. For the 3D‐MRSI scan, only the two‐fold and four‐fold undersampling patterns (constant and variable) using iMUSICAL calibration data were employed. The PI reconstruction was performed for each k_z_‐encoding separately.

A hybrid tt/tk‐GRAPPA reconstruction was employed to reconstruct the undersampled MRSI k‐space. The reconstruction was performed in k‐space before the convolution gridding for the non‐Cartesian MRSI data at hand. The source code available online (https://www.ismrm.org/mri_unbound/sequence.htm) was adapted from radial GRAPPA to concentric‐ring GRAPPA. Each missing k‐space point was reconstructed independently. A segment size of 15 × 4 (points on the ring × rings) was used around each target point for the through‐k‐space GRAPPA part with a kernel size of 3 × 2, i.e., six source points. The kernel was slid through the segment of the calibration data 13 × 3 = 39 times to gather all the through‐k‐space kernel repetitions. The optimal number of calibration time points (i.e., through‐time kernel repetitions) was assessed and found to be 21 points, giving a total of 819 kernel occurrences for determining the GRAPPA weights. In order to compute these weights in an overdetermined manner, the number of kernel occurrences must be at least the number of source points times the number of used channels,^41^ as is true in our case (819 > 6 × 32 = 192).

Two approaches for reconstructing four‐fold and eight‐fold undersampled k‐spaces were compared: a single‐kernel approach (direct reconstruction from R = 4 to R = 1) and a recursive kernel approach. For the latter, rather than performing a direct reconstruction to a fully sampled k‐space, we performed two consecutive two‐fold reconstructions: i.e., the first PI reconstruction yielded a two‐fold undersampled k‐space from a four‐fold undersampled k‐space and the second PI reconstruction finally yielded the desired full k‐space. This procedure was inspired by a recently published study at 9.4T^59^ and the 2D‐GRAPPA‐operator method.^60^ which was found to provide slightly improved reconstructions; see the Results section. The same segment size, kernel size, and calibration repetitions were used as in the single two‐fold reconstruction stated previously. Similarly, in the case of the eight‐fold undersampled data, three consecutive two‐fold reconstructions were performed.

The acquisition of iMUSICAL, “static” aMUSICAL, and “moved” aMUSICAL as calibration data for the different parallel imaging reconstructions was assessed via statistical analysis of SNR and line width [i.e., full width at half maximum (FWHM)] of tCr, as well as CRLBs of metabolites (tCr and tCho) and g‐factors. The g‐factor describing the additional SNR loss caused by imperfect PI reconstruction is defined as:

$$g=\frac{{\mathrm{SNR}}_{\mathrm{FULL}}}{{\mathrm{SNR}}_{\mathrm{PI}}\sqrt{R_{\mathrm{Total}}}}$$

The similarities of PI‐accelerated metabolic maps to the fully sampled ground truth were assessed quantitatively via percent root mean square error (%RMSE) [as calculated in reference 13]. While for iMUSICAL the fully sampled maps coil combined with iMUSICAL were taken as the gold standard, the fully sampled maps coil combined with “static” aMUSICAL were taken as gold standard for both “static” and “moved” aMUSICAL maps. Residual lipid aliasing was assessed by integrating the spectra that were normalized to the median tCr of the fully sampled maps (to account for differences in spectral scaling due to different calibration datasets) in the range of 0.2 to 2.3 ppm. Note that always the same calibration data were used for both PI reconstruction and coil combination.

## 3 RESULTS

### 3.1 Part 1 – coil combination

Among the three types of calibration data used for coil combination, iMUSICAL yielded the highest average SNR values over all volunteers of 21.3 ± 3.7 (Table 1). There were significant intergroup differences among the three coil combination methods (*p* < .001). The mean SNR of iMUSICAL was of 8.8% (*p* = .049, effect size *d* = 0.7) and 9.1% (*p* = .014, *d* = 0.5) higher compared to “static” and “moved” aMUSICAL, respectively.

**Table 1:** Spectral fitting metrics (SNR, FWHM, and metabolite CRLBs) averaged over all five volunteers

	SNR [a.u.]	FWHM [Hz]	tCr CRLB [%]	tCho CRLB [%]	tNAA CRLB [%]
iMUSICAL	21.3 ± 3.7 (21.8)	13.5 ± 2.4 (13.5)	3.7 ± 0.5 (4)	3.9 ± 0.4 (4)	2.1 ± 0.3 (2)
aMUSICAL static	19.6 ± 3.4 (19.4)	13.6 ± 2.5 (13.5)	3.8 ± 0.5 (4)	3.9 ± 0.4 (4)	2.2 ± 0.4 (2)
aMUSICAL moved	19.5 ± 3.3 (19.3)	13.6 ± 2.6 (13.6)	3.9 ± 0.6 (4)	3.9 ± 0.4 (4)	2.2 ± 0.4 (2)

Note

Mean values with standard deviations and medians in brackets are provided. The best results were obtained using iMUSICAL. The differences between iMUSICAL and either of the two aMUSICAL tests were larger than among the two aMUSICAL approaches.

Abbreviations: CRLBs, Cramer‐Rao lower bounds; FWHM, full width at half maximum; SNR, signal‐to‐noise ratio; tCho, total choline; tCr, total creatine; TNAA, total N‐acetyl‐aspartate.

The FWHM values were similar for all three coil combination methods with insignificant intergroup differences (*p* = .9), with mean values of around 13.5 Hz (Table 1). Also, CRLB values showed no significant group differences, but were lowest for iMUSICAL, followed by “static” aMUSICAL and “moved” aMUSICAL (Table 1). For iMUSICAL, the average CRLBs for tCr, tCho, and tNAA were 3.7 ± 0.5, 3.9 ± 0.4, and 2.1 ± 0.3, respectively.

Supporting Information Figure S1 shows sample spectra for volunteer 1 for the three coil combination methods from three different brain regions.

All three coil combination approaches yielded comparable amounts of reliably fitted voxels, with less than 1% difference between iMUSICAL and either aMUSICAL approaches.

**Figure 3:**
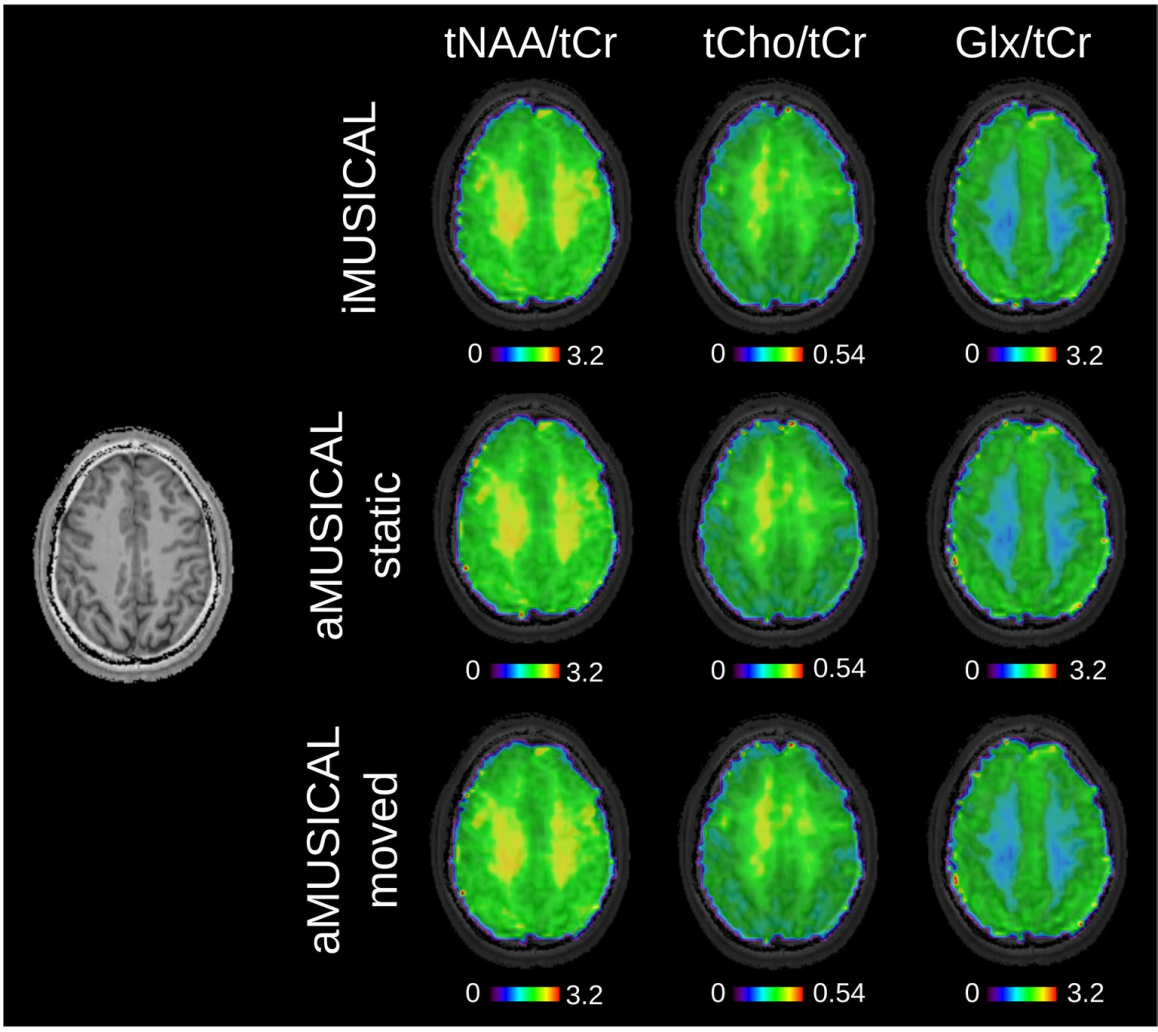
Metabolic ratio maps of volunteer 3. tNAA/tCr, tCho/tCr, and Glx/tCr maps are presented for the three different coil combination approaches. TR: 600 ms, acquisition delay: 1.3 ms, 64 × 64 matrix interpolated to 128 × 128 for display. Very similar metabolic contrasts can be observed among the different coil combinations Glx/tCr, glucose/total creatine; tCho/tCr, total choline/total creatine; tNAA/tCr, total N‐acetyl‐aspartate/total creatine

Metabolic maps of tNAA, tCr, tCho, and Glx are shown in Supporting Information Figure S2 for volunteer 2. A visible variation in metabolite contrast can be observed between iMUSICAL and both “static” and “moved” aMUSICAL. In contrast, metabolic ratio maps of volunteer 3 in Figure 3 display fewer contrast variations between coil combination methods on a qualitative and quantitative basis.

### 3.2 Part 2 – non‐Cartesian through‐time/through‐k‐space GRAPPA

The accuracy of the tt/tk‐GRAPPA reconstruction as a function of time points in the calibration data is shown in Supporting Information Figure S3A. The optimal (i.e., the lowest RMSE) number of temporal calibration points was 21. No further improvement in GRAPPA reconstruction quality was observed with more time points. Supporting Information Figure S3B shows the reconstruction results when performing a direct GRAPPA reconstruction for R = 4 versus our proposed recursive reconstruction of performing two consecutive R = 2 reconstructions. The RMSE values were almost identical, with a small advantage of the recursive reconstruction. Thus, the recursive approach was chosen for all other PI reconstructions.

**Figure 4:**
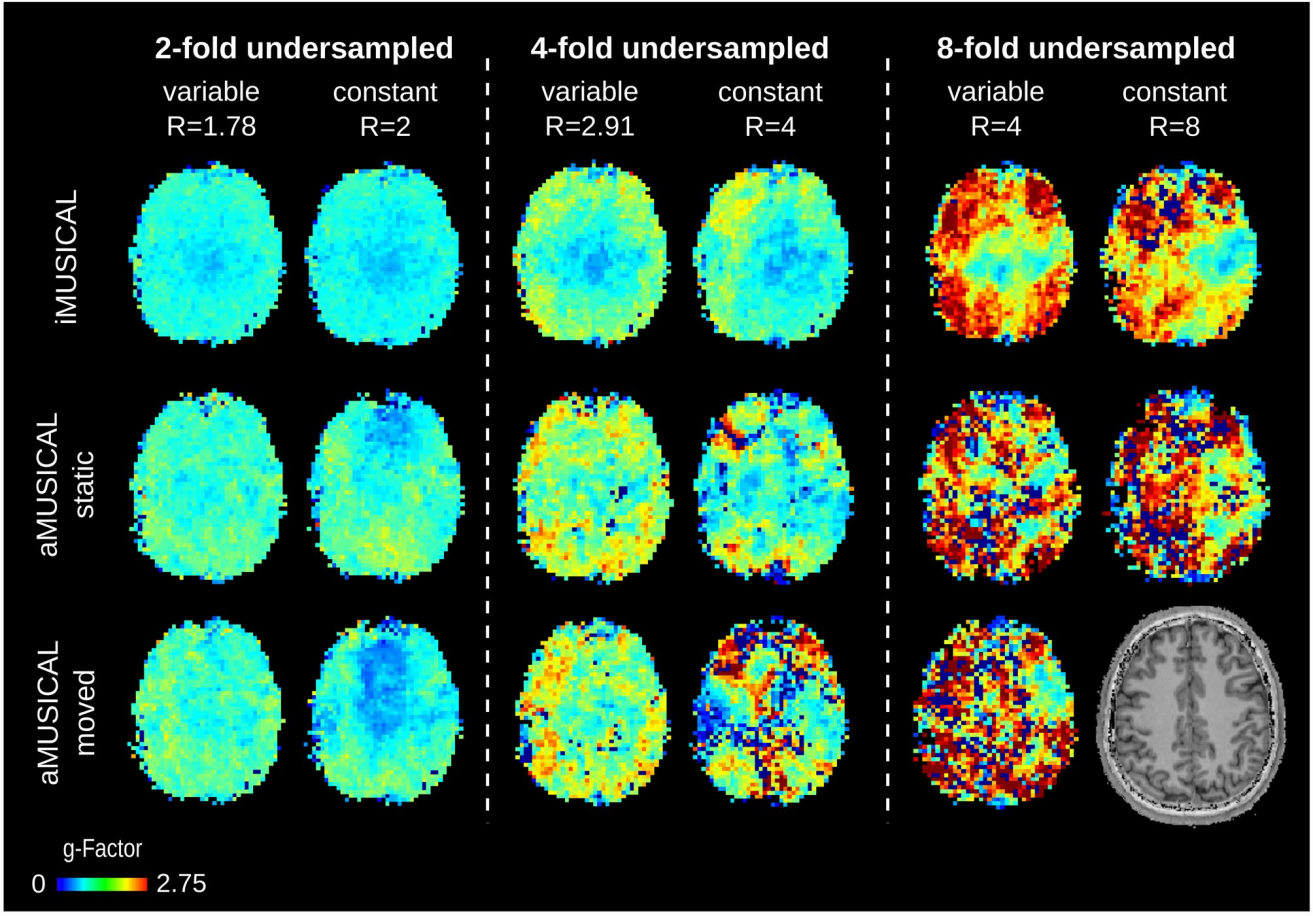
g‐Factor maps are shown for volunteer 2 for different acceleration factors and calibration data. Overall better g‐factors were obtained using iMUSICAL as calibration data. No map could be reconstructed for eight‐fold constant‐density undersampling for “moved” aMUSICAL

Table 2 summarizes the g‐factors obtained for the various acceleration patterns for the three calibration datasets, as well as the CRLB values for tCr and tCho. Overall, iMUSICAL featured the best, i.e., lowest, g‐factors followed by “static” and “moved” aMUSICAL. Mean g‐factors ≤1.07 were recorded for iMUSICAL in both two‐fold undersampling patterns, while a significant 11% increase was obtained for aMUSICAL compared to iMUSICAL (*p* < .05). The g‐factor maps for volunteer 2 are shown in Figure 4.

**Table 2:** g‐Factors and CRLB values for tCr and tCho averaged over all five volunteers listed for the different PI accelerations and for each calibration dataset

	g‐Factors [a.u.]			
	2‐Fold undersampled		4‐Fold undersampled	
	Variable (R = 1.78)	Constant (R = 2)	Variable (R = 2.91)	Constant (R = 4)
iMUSICAL	1.07 ± 0.11 (1.07)	1.03 ± 0.11 (1.03)	1.27 ± 0.23 (1.28)	1.19 ± 0.22 (1.19)
aMUSICAL static	1.19 ± 0.15 (1.19)	1.13 ± 0.23 (1.12)	1.48 ± 0.36 (1.45)	1.29 ± 0.47 (1.23)
aMUSICAL moved	1.19 ± 0.16 (1.19)	1.14 ± 0.29 (1.13)	1.50 ± 0.39 (1.46)	1.42 ± 0.59 (1.34)
	**tCr CRLB [%]**			
	**2‐Fold undersampled**		**4‐Fold undersampled**	
	**Variable (R = 1.78)**	**Constant (R = 2)**	**Variable (R = 2.91)**	**Constant (R = 4)**
iMUSICAL	4.7 ± 2.1 (4)	4.7 ± 2.0 (4)	5.7 ± 2.3 (5)	9.5 ± 3.3 (9)
aMUSICAL static	5.5 ± 2.1 (5)	6.1 ± 2.2 (5)	6.4 ± 2.4 (6)	8.3 ± 2.8 (8)
aMUSICAL moved	5.6 ± 2.1 (5)	6.5 ± 2.8 (6)	8.8 ± 3.0 (8)	10.5 ± 3.6 (10)
	**tCho CRLB [%]**			
	**2‐Fold undersampled**		**4‐Fold undersampled**	
	**Variable (R = 1.78)**	**Constant (R = 2)**	**Variable (R = 2.91)**	**Constant (R = 4)**
iMUSICAL	5.4 ± 2.4 (5)	5.5 ± 2.5 (5)	6.4 ± 2.7 (6)	7.1 ± 2.7 (6)
aMUSICAL static	6.3 ± 2.5 (6)	7.3 ± 2.5 (7)	9.4 ± 3.0 (9)	10.9 ± 3.5 (10)
aMUSICAL moved	6.3 ± 2.4 (6)	7.5 ± 3.1 (6)	9.9 ± 3.2 (9)	12.0 ± 3.7 (11)

Note

Mean values with standard deviations and medians in brackets are provided. Overall, the best results were obtained using iMUSICAL, followed by “static” aMUSICAL. Significantly worse values were obtained for “moved” aMUSICAL

Abbreviations: CRLBs, Cramer‐Rao lower bounds; tCho, total choline; tCr total creatine

Improved CRLB values were obtained for variable‐density undersampling compared to constant undersampling, with higher improvements for four‐fold undersampling patterns. For most undersampling patterns and both metabolites, iMUSICAL exhibited the lowest CRLB values, followed by “static” and “moved” aMUSICAL. While increases of 17% and 18% in tCr CRLB were obtained for two‐fold variable‐density undersampling for “static” and “moved” aMUSICAL, respectively, compared to iMUSICAL (*p* < .05), up to 53% and 69% increases in tCho CRLB were found for four‐fold constant undersampling (*p* < .05). Overall, a significant degradation in CRLB values that increased with higher acceleration factors was obtained for the “moved” aMUSICAL calibration data.

These results are supported by Supporting Information Figure S4, where the CRLB maps of tCr are depicted for volunteer 2. While for the fully sampled maps, only minor differences were visible (as reported previously in the coil combination results), the superior performance of iMUSICAL became increasingly pronounced with increasing acceleration factors.

**Figure 5:**
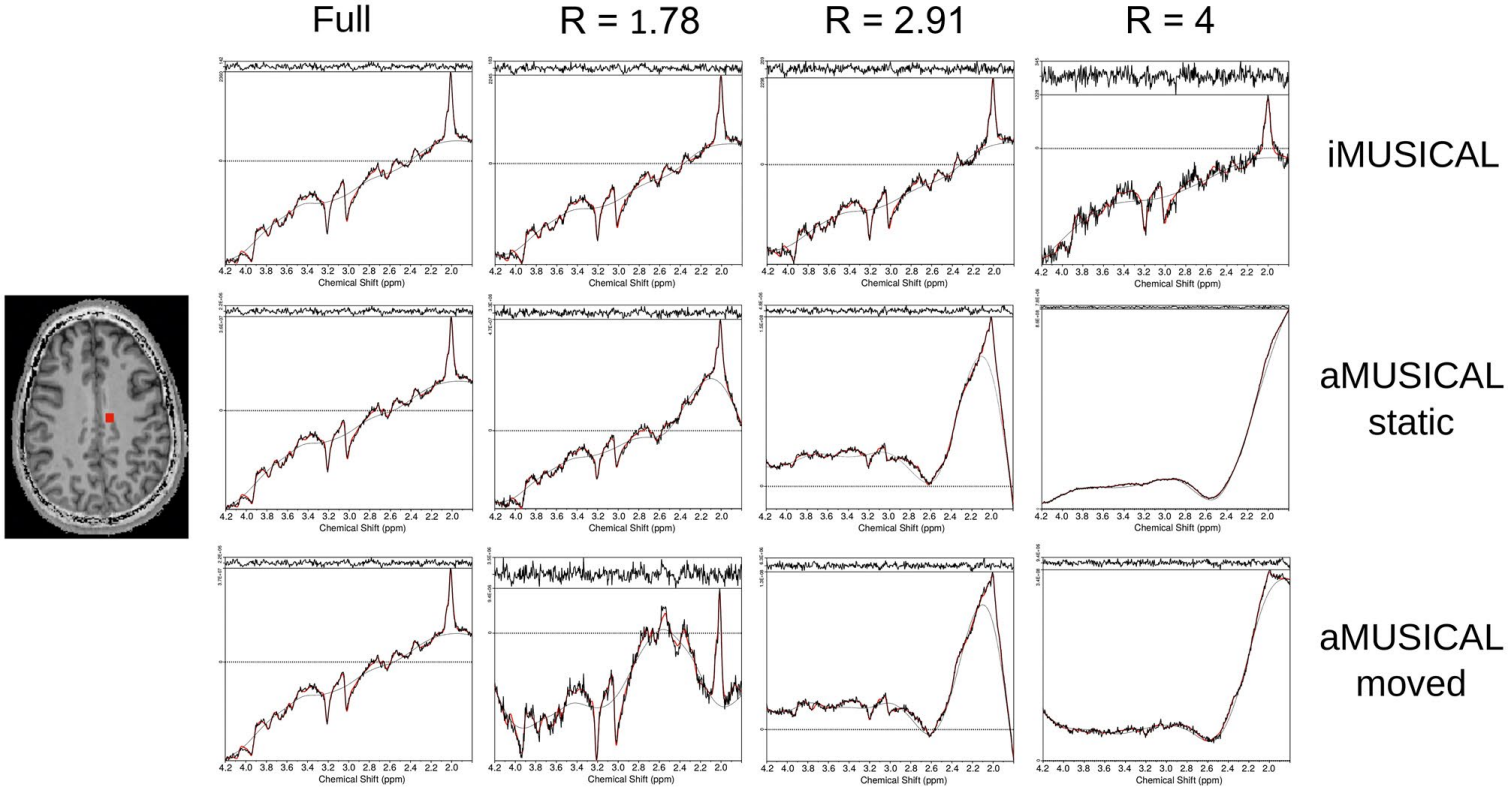
Spectra for volunteer 2 from one representative voxel position for all variable‐density PI reconstructions, including the fully sampled ground‐truth spectra. High‐quality spectra were obtained for iMUSICAL, even at high acceleration factors. Hardly any lipid artifacts were visible when using iMUSICAL, whereas both aMUSICAL cases showed strong lipid artifacts. “Moved” aMUSICAL also experienced a significant spectral SNR loss. PI, parallel imaging; SNR, signal‐to‐noise ratio

Figure 5 shows sample spectra for volunteer 2 from one representative voxel position for all variable‐density PI reconstructions, including the fully sampled ground‐truth spectra. While iMUSICAL showed no lipid artifacts in the upfield spectral part, strong lipid artifacts can be observed for both aMUSICAL cases, with an additional decrease in SNR.

**Figure 6:**
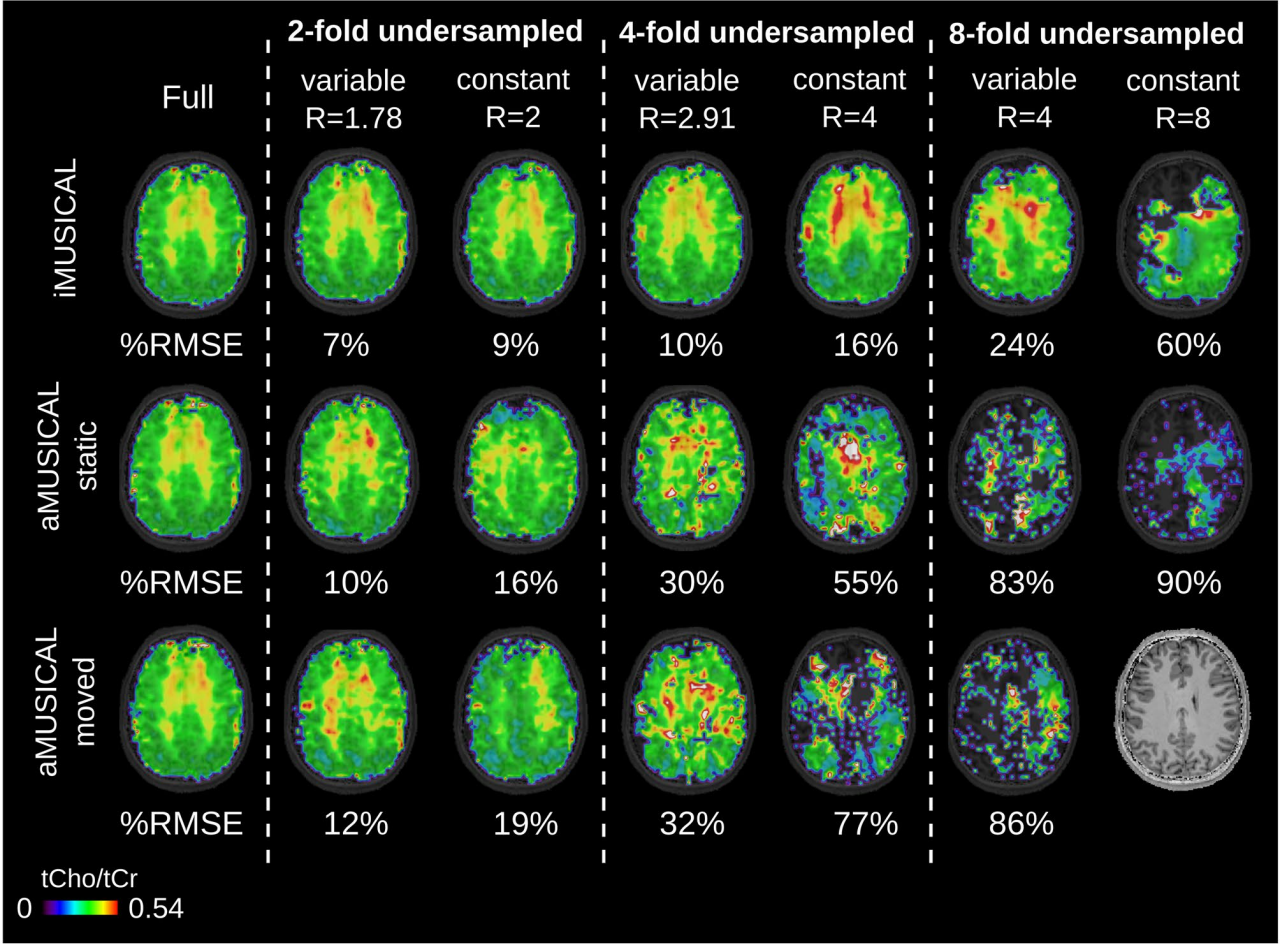
Metabolic ratio maps (tCho/tCr) of volunteer 2 are presented for the different PI acceleration factors and calibration datasets, including %RMSE to the fully sampled ground‐truth. TR: 600 ms;, acquisition delay: 1.3 ms; 64 × 64 matrix interpolated to 128 × 128 for display. Using the interleaved iMUSICAL as calibration yielded significantly improved PI reconstructions compared to both aMUSICAL datasets. No map could be reconstructed for eight‐fold constant‐density undersampling for “moved” aMUSICAL PI, parallel imaging; %RMSE; percent root mean square error; tCho/tCr, total choline/total creatine; TR,repetition time

The metabolic ratio maps (tCho/tCr) are shown in Figure 6 for volunteer 2 for all PI reconstructions, including the fully sampled ground‐truth and RMSE. A high similarity to the fully sampled map can be observed for maps with up to four‐fold variable‐density undersampling (RSME ≤ 10%) in the case of iMUSICAL, while “static” aMUSICAL already exhibited noticeable contrast variations for two‐fold constant‐density undersampling or higher (RMSE > 30%). Overall, “moved” aMUSICAL showed the poorest results. In the case of eight‐fold variable‐density undersampling, only iMUSICAL allowed for a reasonable map to be reconstructed.

**Figure 7:**
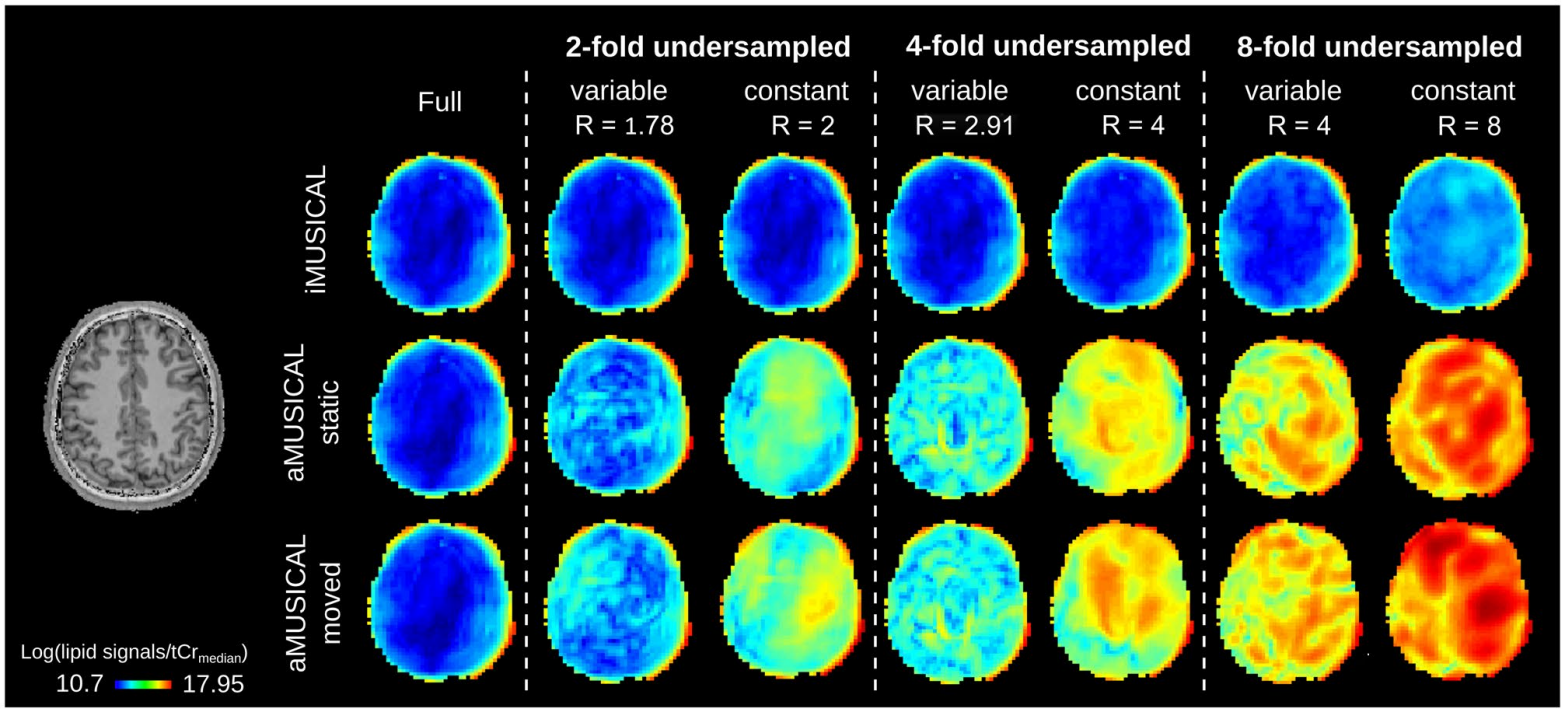
Maps of residual lipid signals of volunteer 4 are presented for the different PI acceleration factors and calibration datasets, including the fully sampled ground‐truth. Residual lipid aliasing was lowest for iMUSICAL even for eight‐fold accelerations, while strong artifacts can be observed even for two‐fold accelerations for both aMUSICAL cases, with “moved” aMUSICAL performing worst PI, parallel imaging

Maps of residual lipid signals are shown in Figure 7 for volunteer 4. A significant increase of residual lipid signals was observed for both aMUSICAL cases compared to iMUSICAL. While residual lipid signals were low for iMUSICAL even for eight‐fold accelerations, strong artifacts can be observed even for two‐fold accelerations for both aMUSICAL cases, with “moved” aMUSICAL performing worst.

**Figure 8:**
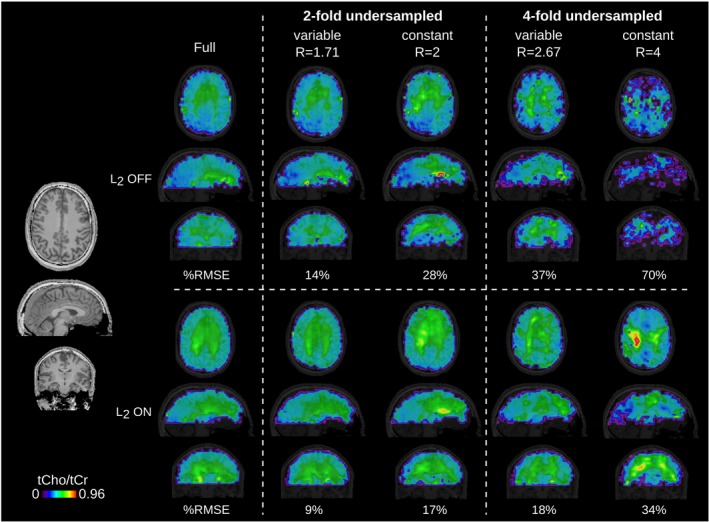
The Tt/tk‐GRAPPA acceleration applied to a 3D stack‐of‐concentric‐rings MRSI dataset, including %RMSE to the fully‐sampled ground‐truth. Metabolic ratio maps of tCho/tCr are shown for volunteer 6 with and without L_2_ lipid regularization for acceleration factors up to 2. TR: 600 ms, acquisition delay: 1.3 ms, 48 × 48 × 31 matrix interpolated to 96 × 96 × 62 for display %MRSI, percent MR spectroscopic imaging; tCho/tCr, total choline/total creatine; TR, repetition time; Tt/tk‐GRAPPA, through‐time/through‐k‐space‐generalized autocalibrating partially parallel acquisition

Figure 8 depicts the feasibility of tt/tk‐GRAPPA for 3D stack‐of‐concentric‐rings MRSI data of volunteer 6. Metabolic ratio maps of tCho/tCr are shown with and without L_2_ lipid regularization for acceleration factors up to 4. A high similarity to the fully sampled maps can be observed for the L_2_‐regularized two‐fold variable‐density undersampled case (RMSE = 9%).

## 4 DISCUSSION

In this work, we presented an interleaved, water‐reference acquisition scheme, termed iMUSICAL, which not only allowed the reference‐coil‐independent combination of multichannel MRSI data, but also served as calibration for PI reconstructions of undersampled MRSI data.

The coil combination performance using iMUSICAL calibration data was evaluated in vivo at 7T compared to the established MUSICAL method for a single‐slice FID‐MRSI sequence. Our results provide evidence that iMUSICAL is superior to adapted MUSICAL considering quality metrics and methodological simplicity.

Furthermore, we presented the first application of tt/tk‐GRAPPA acceleration in a non‐Cartesian MRSI sequence. Again, using iMUSICAL as calibration data for the PI reconstruction proved superior to using aMUSICAL and thus, allowed for higher acceleration factors. A detailed investigation, including motion‐related PI artifacts, was performed in single‐slice FID‐MRSI experiments. Last but not least, the feasibility and benefits of this tt/tk‐GRAPPA acceleration for volumetric 3D‐MRSI were evaluated and will be discussed.

### 4.1 Coil combination

In a first step, we investigated the efficiency of using our proposed iMUSICAL data as calibration data for coil combination compared to the established MUSICAL method. Although several other reference‐coil‐independent coil combination approaches exist,^61^, ^62^, ^63^, ^64^ we have focused solely on a comparison of the iMUSICAL method to the MUSICAL method that has been shown to perform well at 7T and with FID‐based sequences, where a higher spatial phase variation is encountered.^19^ Strasser et al. previously compared MUSICAL to two established methods and found an increase in SNR, a decrease in CRLB values, and improved spectral quality compared to the method of Brown et al.^62^ In comparison to using sensitivity maps, the SNR was increased, the computational and hardware demands decreased, and the resulting spectra were prephased.^19^ Thus, building on these published results, our proposed interleaved iMUSICAL was compared only to MUSICAL, focusing on iMUSICAL's versatile application to PI as well as its robustness to motion.

Compared to the original MUSICAL coil combination, iMUSICAL can be tailored to any spatial‐spectral encoding trajectory, allowing for significant acceleration factors of up to two orders of magnitude^65^, ^66^ necessary for whole‐brain MRSI at 7T. Furthermore, in contrast to the aMUSICAL prescan, the interleaved acquisition of iMUSICAL adds no scan time to the MRSI measurement. This may not be critical for 2D‐MRSI, but for whole‐brain MRSI the difference is noticeable (e.g., ~2 to 3 min).

In addition to comparing iMUSICAL‐based and aMUSICAL‐based coil combinations, the effects of head motion were assessed. All three coil combination approaches, using either iMUSICAL, “static,” or “moved” aMUSICAL calibration data, yielded comparable fitting quality results (SNR, FWHM, and CRLBs), with slightly improved results using iMUSICAL data. Metabolic map qualities were very similar, with hardly any visible differences. The differences between iMUSICAL and either of the aMUSICAL methods were more pronounced than the differences between the two aMUSICAL methods. Although motion certainly has a significant effect on MRSI data quality,^67^ all our results suggest that coil combination itself is very robust (at least when using metabolite ratios), while other postprocessing steps (such as PI reconstruction, as shown later) are quite sensitive to motion.

The coil combinations evaluated showed some contrast differences in the unnormalized metabolic maps, which were probably introduced by saturation effects of the respective calibration data. While for the rapid aMUSICAL prescan, a TR of 50 ms was selected, the iMUSICAL data were acquired with an effective TR of 600 ms, as dictated by the MRSI sequence due to iMUSICAL's interleaved acquisition approach. This introduced slightly different contrasts, which disappeared entirely for metabolite ratio maps. This is not a severe limitation, since, in clinical MRS, metabolite values are reported relative to other metabolites (e.g., tCho/tNAA) or water signal.

While MRSI methods without water suppression (e.g., metabolic cycling^17^ or SPICE^18^) provide a water reference image right away, previous reports have already described the implementation of interleaved water‐reference‐acquisition schemes in water suppressed MRSI sequences. Ebel et al. used nonlocalized water reference information only for real‐time frequency updating,^68^ while Maudsley et al. used localized water acquisitions for multichannel combination in an echo‐planar spectroscopic imaging sequence.^69^ However, the latter report addresses coil combination only as a short remark and there was no systematic evaluation of the feasibility and performance of interleaved water scans for coil combination compared to the more widely used noninterleaved, water‐reference prescans. Moreover, our iMUSICAL approach differs from these two reports in that discrepancies between MRSI and prescan data are minimized by mimicking the acquisition and readout scheme of the MRSI sequence in the interleaved iMUSICAL water reference scan.

### 4.2 Non‐Cartesian through‐time/through‐k‐space GRAPPA

In a second step, we presented the first implementation and assessment of tt/tk‐GRAPPA reconstructions for non‐Cartesian MRSI encoding. The proof of principle was evaluated for 2D‐MRSI, because non‐Cartesian PI is expected to achieve superior g‐factors compared to Cartesian PI (i.e., here in z‐dimension).^70^ Although GRAPPA acceleration is not necessary for 2D concentric ring MRSI with multiple averages, as it is always better to accelerate by reducing the number of averages than by using GRAPPA, our goal was to establish tt/tk‐GRAPPA for 3D‐MRSI. To mimic a similar SNR to that in 3D‐MRSI without the drawbacks of long measurement and processing times, we chose to show the performance of tt/tk‐GRAPPA for 2D‐MRSI with multiple averages. The results can be directly transferred to tt/tk‐GRAPPA for 3D‐MRSI, since we provide a 3D example, and the 3D reconstruction is performed partitionwise. The Tt/tk‐GRAPPA was initially demonstrated for real‐time 2D and 3D cardiac MRI.^40^, ^41^ where acquisition times must be ~50 ms to capture cardiac motion adequately. To achieve both high spatial and temporal resolutions in dynamic cardiac imaging, Seiberlich et al. combined acceleration techniques based on non‐Cartesian, radial k‐space trajectories and tt/tk‐GRAPPA‐based parallel imaging. In this regard, MRSI is similar to cardiac MRI, as it also acquires a rapid time series of images (i.e., the temporal FID dimension) and greatly benefits from the aforementioned acceleration techniques. While both techniques per se (non‐Cartesian readout and parallel imaging) are well‐established tools in MRSI, this report marks the first combined implementation of these two techniques, theoretically enabling the highest acceleration factors possible compared to conventional MRSI.

As described by Seiberlich et al^41^ the inherent difficulties in finding multiple repetitions of calibration kernels in k‐space that are similar can be mitigated by collecting the calibration information from small segments and multiple repetitions in time (i.e., using a through‐time/through‐k‐space kernel). In a nutshell, the advantages of tt/tk‐GRAPPA include its relative independence of the chosen k‐space trajectories, the fact that calibration weights are also determined from the time dimension, and fewer assumptions on k‐space similarities need to be made. However, disadvantages might arise in peripheral k‐space regions with low SNR, where the weights might be determined imprecisely.

Overall, using the tt/tk‐GRAPPA technique on concentric‐ring MRSI data achieved promising results, especially with the interleaved iMUSICAL calibration data. Compared to the “static” aMUSICAL, more robust PI reconstructions, lower g‐factors, lower CRLBs, less residual lipid aliasing, and metabolic maps of higher quality could be achieved with iMUSICAL. Spectral quality with iMUSICAL was high, even for high accelerations with minimal residual lipid artifacts, whereas both aMUSICAL cases showed significant lipid aliasing in the upfield spectral part.

It is well known that any mismatch between calibration and imaging data, e.g., due to motion, causes erroneous PI reconstructions.^57^, ^71^ This was investigated by an aMUSICAL prescan acquired with a rotated head position. Expected overall worse PI reconstruction results were obtained with lower spectral quality, lower SNR, lower g‐factors, and higher CRLB values. All our results provide evidence that acquiring calibration data in an interleaved fashion with the proposed iMUSICAL is highly beneficial compared to a prescan measured before the MRSI scan due to the close temporal proximity of acquiring calibration and MRSI data. Since the g‐factors in GRAPPA explicitly depend on the calibration data through the GRAPPA weights,^72^ smaller g‐factors are expected when more suitable calibration data are used. This is in agreement with MRI reports.^43^, ^73^, ^74^, ^75^

Many previous reports have highlighted the benefits of variable‐density undersampling with respect to PI reconstruction artifacts over constant‐density undersampling, as the inner k‐space contains low spatial frequencies that are responsible for the main image contrast, while the outer k‐space, with its high spatial frequencies, determines mainly detailed lines and edges.^26^, ^59^ In this report, constant‐density undersampling is compared to a simple variable‐density pattern characterized by full sampling of the innermost five rings followed by constant‐density undersampling of the outer k‐space. Although a direct comparison between constant‐undersampling and variable‐density undersampling with identical effective acceleration was not possible, our results show a clear trend that a variable‐density undersampling yields more robust and artifact‐reduced reconstructions, which may not only be reflected in significantly lower CRLB values, but also in increased spectral and metabolic map quality. In addition to improved PI reconstructions, variable‐density undersampling offers another benefit. Wilson et al. proposed a variable, temporal interleave acceleration scheme (note: in this report constant temporal interleaves were used) for concentric‐ring trajectories, in which only the minimum amount of required temporal interleaves within gradient limitations for each ring were acquired.^76^ Thus, inner rings are measured with one temporal interleaf, while the outermost rings are measured with three temporal interleaves for a 3.4 × 3.4 mm^2^ inplane resolution, which led to 37% faster scans. More importantly, the variable‐density undersampling approach would significantly improve the trade‐off between the acceleration factor and reconstruction quality, as fully sampling the most contrast‐relevant inner five rings is negligible, with regard to time, compared to the higher temporal interleaf numbers in the k‐space periphery where undersampling would then apply. In view of the very promising results obtained for two‐fold or four‐fold variable‐density undersampling in this study, and its potential further improvement using variable temporal interleaves, this appears to be the most attractive option.

Previous phase‐encoded MRSI reports that have used, for example, (2+1)D‐Controlled Aliasing In Parallel Imaging Results IN Higher Acceleration with an optimized undersampling pattern,^13^ or MultiNet PyGRAPPA with machine‐learning‐based reconstructions,^59^ achieved higher PI acceleration factors (up to 10) than those shown here. However, since we already highly accelerate using the non‐Cartesian concentric‐ring trajectory, we are mainly limited by SNR, tolerating only moderate additional accelerations via PI. This is in agreement with Tsai et al.^32^ and Zhu et al.,^33^ who also used conservative PI acceleration factors in their echo‐planar spectroscopic imaging sequences. Compared to accelerating only with PI, the moderate PI acceleration factors used in this report kept lipid aliasing artifacts at a minimum level and rendered them negligible for two‐fold variable‐density undersampling.

We qualitatively demonstrated the feasibility of tt/tk‐GRAPPA for whole‐brain 3D‐MRSI data with highly promising results for moderate accelerations (i.e., two‐fold variable‐density undersampling). Combined with the aforementioned variable temporal interleaf approach, clinically attractive scan times of <5 min for 3.4 × 3.4 × 3.4 mm^3^ nominal voxel sizes can be achieved.

### 4.3 Further comments on the general application of iMUSICAL

In principle, iMUSICAL can also be applied to spin‐echo MRSI, but might lead to higher specific absorption rate demands if the full spin‐echo pulse series is replicated in the iMUSICAL prescan. Alternatively, an FID‐based iMUSICAL can be incorporated as shown for a 1D‐semiLASER sequence.^77^

The multiple trajectory repetitions at different TEs of the iMUSICAL prescan could additionally be used to obtain *B*_0_ maps. Ultimately, this would provide MRSI data with intrinsically corrected 0th‐order, frequency‐shift errors, and eddy‐current errors. This would lead to substantial acceleration and reliability of spectral fitting via, e.g., LCModel. The data could be further used to scale the MRSI data to water.^69^

### 4.4 Limitations

Motion‐related artifacts were only studied by a mismatched global prescan (i.e., “moved” aMUSICAL). No head rotation was conducted during the MRSI scan, as this would also affect the quality of the MRSI data themselves, thereby rendering a comparison to the fully sampled ground‐truth impossible. The robustness of the tt/tk‐GRAPPA accelerated FID‐MRSI presented here could be further improved by implementing an image‐based navigator for real‐time motion correction and dynamic frequency/shim updates,^78^, ^79^ thus also eliminating any artifacts that arise from motion during the MRSI acquisition.

Furthermore, the originally proposed MUSICAL (i.e., prescan based on Cartesian gradient echo imaging) was not directly compared to iMUSICAL, because the mismatch in spatial response functions due to different gradient trajectories would likely introduce additional systematic errors.

Only a narrow spectral range from 1.8 ppm to 4.2 ppm was fitted. This prevents the quantification of certain metabolites (e.g., lactate) but reduces sensitivity to potential lipid contamination.

As we retrospectively undersampled the MRSI k‐space for this study, fully sampled calibration datasets were available, as well as fully sampled MRSI k‐spaces that served as ground truth for comparisons of GRAPPA reconstruction quality. Because of iMUSICAL's interleaved acquisition, omitting every other ring (for R = 2) would, however, result in missing calibration data in a prospectively undersampled case. For R = 2, this could be resolved by immediately acquiring two consecutive iMUSICAL modules, spoiling the residual transverse magnetization in between. The applicability of this consecutive approach to R > 2 accelerations would need further optimization considering saturation effects, but for our purposes R **≤** 2 seems most promising with an attractive compromise among moderate acceleration, lowest g‐factor penalty, and negligible reconstruction artifacts.

## 5 CONCLUSION

Non‐Cartesian, tt/tk‐GRAPPA is feasible for 2D and 3D‐MRSI data and can further boost the already‐high acceleration provided by SSE‐based, concentric‐ring trajectories. Promising results have been achieved using iMUSICAL, an interleaved, water‐acquisition scan, as a source of multipurpose reference data. iMUSICAL proved to be a flexible and robust approach for the efficient coil combination of MRSI data under challenging conditions (i.e., *B*_0 _≥ 7T, many coil elements, no reference coil, low SNR, possible spectral artifacts, motion/instability‐related artifacts, first‐order phase errors) and served as calibration data for PI reconstructions, reducing their susceptibility to subject motion and scanner instability‐related artifacts.

## Supporting information


**FIGURE S1** Representative spectra for volunteer 1 in three voxel positions (central white matter, temporal gray matter, and frontal gray matter). Similar results were obtained throughout the individual coil combinations, with slightly less SNR in the “moved” aMUSICAL case SNR, signal‐to‐noise ratio
**FIGURE S2** Metabolic maps of volunteer 2. tNAA, tCr, tCho, and Glx maps are presented for the three different coil combination approaches. TR: 600 ms; acquisition delay: 1.3 ms; 64 × 64 matrix interpolated to 128 × 128 for display. Differences in contrast were observed between iMUSICAL and aMUSICAL, while only subtle differences were apparent between both aMUSICAL results Glx, glucose; tCho, total choline; tCr, total creatine; tNAA, total N‐acetyl‐aspartate; TR, repetition time
**FIGURE S3** A, Accuracy of the tt/tk‐GRAPPA reconstruction (measured as RMSE to the fully sampled ground truth) as a function of time points in the calibration data as well as overdetermination of the GRAPPA reconstruction. The dashed line indicates the value used in this study; B, Comparison between a recursive GRAPPA reconstruction (from R = 4 to R = 2, then from R = 2 to R = 1) as used in this study and a single‐kernel approach (from R = 4 to R = 1). Slightly improved RMSE were obtained for the recursive approach RMSE, root mean square error; tt/tk‐GRAPPA, through‐time/through‐k‐space‐generalized autocalibrating partially parallel acquisition
**FIGURE S4** CRLB maps of tCr are depicted for volunteer 2 for different acceleration factors and calibration data. White colored voxels indicate CRLB values of >20%. No map could be reconstructed for eight‐fold constant‐density undersampling CRLB, Cramer‐Rao lower bound; tCr, total creatine
