# Supplementary material for: Non‐Cartesian GRAPPA and coil combination using interleaved calibration data – application to concentric‐ring MRSI of the human brain at 7T
Source: Magn Reson Med. 2019 Jun 10;82(5):1587–603. doi: 10.1002/mrm.27822 (PMC6772100; doi:10.1002/mrm.27822)
Supplement: Supplementary file 1 — FIGURE S1 Representative spectra for volunteer 1 in three voxel positions (central white matter, temporal gray matter, and frontal gray matter). Similar results were obtained throughout the individual coil combinations, with slightly less SNR in the “moved” aMUSICAL case SNR, signal‐to‐noise ratio FIGURE S2 Metabolic maps of volunteer 2. tNAA, tCr, tCho, and Glx maps are presented for the three different coil combination approaches. TR: 600 ms; acquisition delay: 1.3 ms; 64 × 64 matrix interpolated to 128 × 128 for display. Differences in contrast were observed between iMUSICAL and aMUSICAL, while only subtle differences were apparent between both aMUSICAL results Glx, glucose; tCho, total choline; tCr, total creatine; tNAA, total N‐acetyl‐aspartate; TR, repetition time FIGURE S3 A, Accuracy of the tt/tk‐GRAPPA reconstruction (measured as RMSE to the fully sampled ground truth) as a function of time points in the calibration data as well as overdetermination of the GRAPPA reconstruction. The dashed line indicates the value used in this study; B, Comparison between a recursive GRAPPA reconstruction (from R = 4 to R = 2, then from R = 2 to R = 1) as used in this study and a single‐kernel approach (from R = 4 to R = 1). Slightly improved RMSE were obtained for the recursive approach RMSE, root mean square error; tt/tk‐GRAPPA, through‐time/through‐k‐space‐generalized autocalibrating partially parallel acquisition FIGURE S4 CRLB maps of tCr are depicted for volunteer 2 for different acceleration factors and calibration data. White colored voxels indicate CRLB values of >20%. No map could be reconstructed for eight‐fold constant‐density undersampling CRLB, Cramer‐Rao lower bound; tCr, total creatine [file MRM-82-1587-s001.docx]

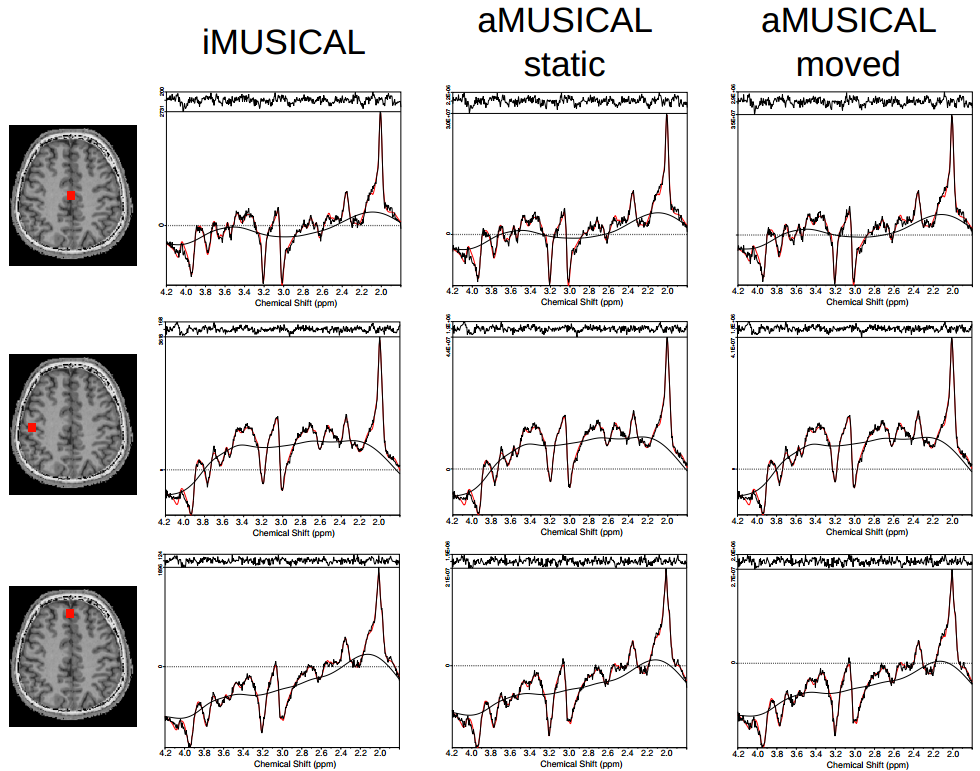


Supporting Information Figure S1: Representative spectra for volunteer 1 in three voxel positions (central white matter, temporal gray matter, and frontal gray matter). Similar results were obtained throughout the individual coil combinations, with slightly less SNR in the “moved” aMUSICAL case.


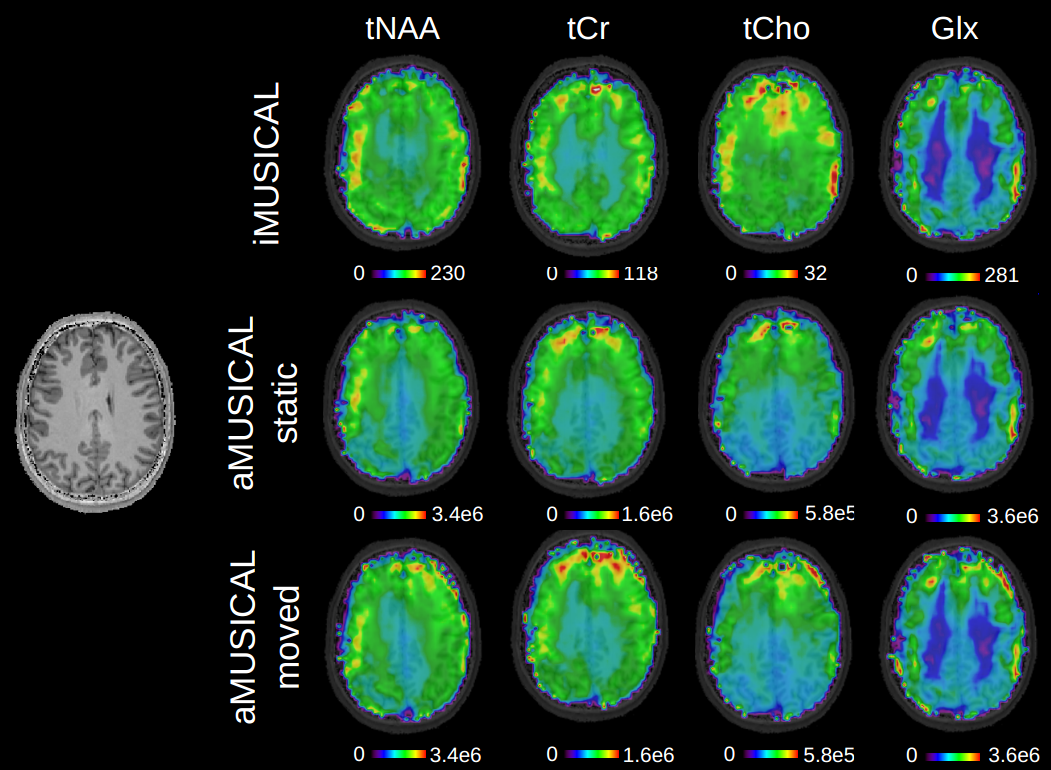


Supporting Information Figure S2: Metabolic maps of volunteer 2. tNAA, tCr, tCho, and Glx maps are presented for the three different coil combination approaches. TR: 600 ms, acquisition delay: 1.3 ms, 64×64 matrix interpolated to 128×128 for display. Differences in contrast were observed between iMUSICAL and aMUSICAL, while only subtle differences were apparent between both aMUSICAL results.


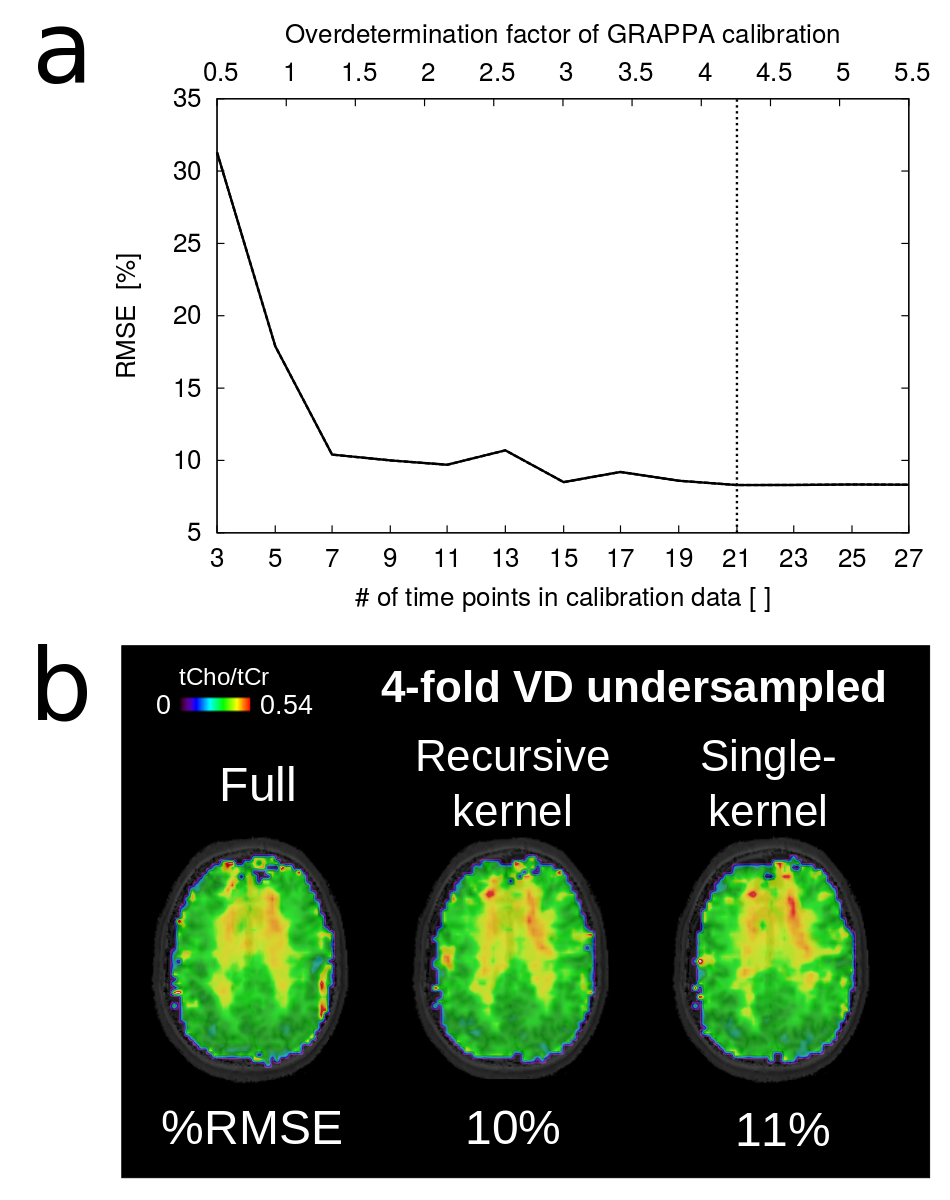


Supporting Information Figure S3: a) Accuracy of the tt/tk-GRAPPA reconstruction (measured as RMSE to the fully-sampled ground truth) as a function of time points in the calibration data as well as overdetermination of the GRAPPA reconstruction. The dashed line indicates the value used in this study; b) Comparison between a recursive GRAPPA reconstruction (from R=4 to R=2, then from R=2 to R=1) as used in this study and a single-kernel approach (from R=4 to R=1). Slightly improved RMSE were obtained for the recursive approach.


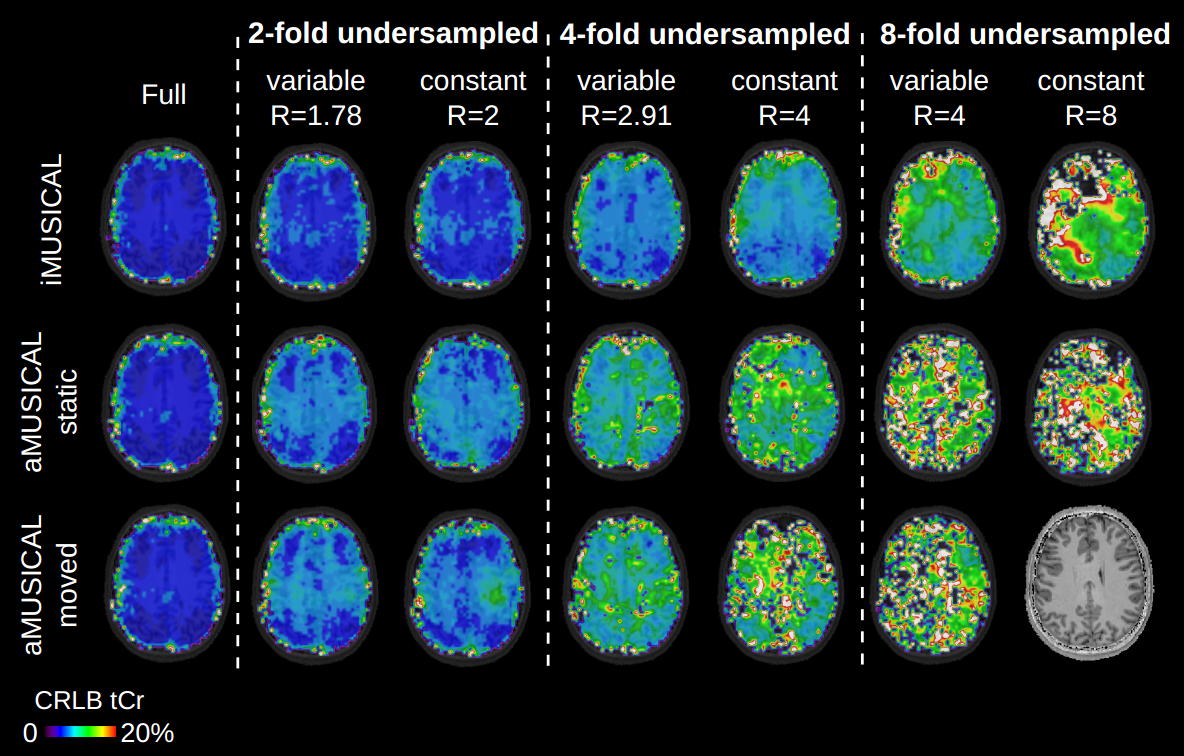


Supporting Information Figure S4: CRLB maps of tCr are depicted for volunteer 2 for different acceleration factors and calibration data. White colored voxels indicate CRLB values of >20%. No map could be reconstructed for constant eight-fold undersampling.
